# Portable, low-field magnetic resonance imaging enables highly accessible and dynamic bedside evaluation of ischemic stroke

**DOI:** 10.1126/sciadv.abm3952

**Published:** 2022-04-20

**Authors:** Matthew M. Yuen, Anjali M. Prabhat, Mercy H. Mazurek, Isha R. Chavva, Anna Crawford, Bradley A. Cahn, Rachel Beekman, Jennifer A. Kim, Kevin T. Gobeske, Nils H. Petersen, Guido J. Falcone, Emily J. Gilmore, David Y. Hwang, Adam S. Jasne, Hardik Amin, Richa Sharma, Charles Matouk, Adrienne Ward, Joseph Schindler, Lauren Sansing, Adam de Havenon, Ani Aydin, Charles Wira, Gordon Sze, Matthew S. Rosen, W. Taylor Kimberly, Kevin N. Sheth

**Affiliations:** 1 Department of Neurology, Yale School of Medicine, New Haven, CT, USA.; 2 Department of Neurosurgery, Yale School of Medicine, New Haven, CT, USA.; 3 Neuroscience Intensive Care Unit, Yale New Haven Hospital, New Haven, CT, USA.; 4 Department of Emergency Medicine, Yale School of Medicine, New Haven, CT, USA.; 5 Department of Radiology and Biomedical Imaging, Yale School of Medicine, New Haven, CT, USA.; 6 Athinoula A. Martinos Center for Biomedical Imaging, Massachusetts General Hospital, Charlestown, MA, USA.; 7 Department of Neurology, Massachusetts General Hospital, Boston, MA, USA.

## Abstract

Brain imaging is essential to the clinical management of patients with ischemic stroke. Timely and accessible neuroimaging, however, can be limited in clinical stroke pathways. Here, portable magnetic resonance imaging (pMRI) acquired at very low magnetic field strength (0.064 T) is used to obtain actionable bedside neuroimaging for 50 confirmed patients with ischemic stroke. Low-field pMRI detected infarcts in 45 (90%) patients across cortical, subcortical, and cerebellar structures. Lesions as small as 4 mm were captured. Infarcts appeared as hyperintense regions on T2-weighted, fluid-attenuated inversion recovery and diffusion-weighted imaging sequences. Stroke volume measurements were consistent across pMRI sequences and between low-field pMRI and conventional high-field MRI studies. Low-field pMRI stroke volumes significantly correlated with stroke severity and functional outcome at discharge. These results validate the use of low-field pMRI to obtain clinically useful imaging of stroke, setting the stage for use in resource-limited environments.

## Teaser

Portable, bedside, low-field magnetic resonance imaging provides clinically valuable imaging of ischemic stroke.

## INTRODUCTION

Stroke is a leading cause of disability and death that affects 15 million people worldwide each year (*1*–*3*). Brain imaging is essential for confirming clinically suspected stroke, facilitating the implementation of acute treatment and secondary prevention pathways, including medical and neurosurgical interventions (*4*, *5*). Noncontrast computed tomography (NCCT) and magnetic resonance imaging (MRI) are commonly used for the evaluation of stroke, although MRI provides superior visualization of the brain through high-resolution and multimodal imaging (*6*). In traditional imaging pathways, patients must be transported from a controlled clinical environment to remote radiologic suites that contain the stationary NCCT or MRI scanner. Intrahospital transport of patients is associated with numerous cardiovascular and respiratory risks and is often delayed (*7*–*14*), which can limit timely and safe neuroimaging for critically ill patients with stroke. Moreover, these imaging units are costly to purchase, site, and maintain, which restricts the number of available imaging scanners in clinical health centers and can create a bottleneck in the workflow (*15*).

Portable imaging devices offer an avenue to circumvent the disadvantages associated with conventional imaging approaches, as they can be brought directly to the point of care and are less costly than stationary imaging units (*16*). Portable computed tomography (pCT) is a well-explored portable imaging modality that is used in some tertiary and quaternary health care centers to image patients with acute brain injuries (*16*). However, pCT requires lead shielding around the point of care and specialized technicians to operate the device (*17*), which restricts its availability and ease of use. In addition, pCT is associated with ionizing radiation risk for both patients and the pCT operator (*18*–*21*). Furthermore, although NCCT imaging can accurately detect hemorrhage, x-ray technology is limited in its ability to detect acute ischemia compared to MRI (*6*). For these reasons, pCT is not widely used in the clinical management of patients with stroke.

We recently deployed a portable MRI (pMRI) that operates at very low magnetic field strength (0.064 T) to the bedside of intensive care patients as a novel neuroimaging solution (*22*). Similar to high-field MRI scanners, pMRI can acquire T1-weighted (T1W), T2W, fluid-attenuated inversion recovery (FLAIR), and diffusion-weighted imaging (DWI) sequences. Unlike high-field MRI scanners, however, the low-field pMRI is mobile and can operate safely at the patient’s bedside without the use of specialized shielding or projectile risk from nearby ferromagnetic hospital equipment. Moreover, low-field pMRI avoids exposure to ionizing radiation that is associated with NCCT (fixed and portable) and does not require a specialized MRI technician for operation because of its ease of use (*23*).

Low-field pMRI has been shown to be safe and feasible (*22*, *23*). In the context of acute stroke evaluation and acute neurological deterioration, detecting and ruling out intracranial hemorrhage is a cornerstone of neuroimaging (*4*). A prior report suggests clinical utility of low-field pMRI in this setting (*24*). To date, however, there has been scant assessment of this approach for the detection of ischemic stroke, which accounts for 87% of all strokes (*2*). The ability of a bedside device to affirm the presence and evaluate the extent of cerebral infarction can establish a clinical diagnosis, obviating the need for additional procedures and expediting triage and treatment pathways. Similarly, a portable, bedside solution that can facilitate diagnostic confirmation would enable the delivery of care in numerous settings where stroke evaluation is limited, including inpatient units, mobile vehicles (*25*), and low-resource settings (*26*).

In the present work, we provide the first systematic evaluation of pMRI for imaging cerebral infarction and its clinical utility in the management of ischemic stroke. We used pMRI to obtain bedside head imaging of patients with ischemic stroke in the emergency department (ED), inpatient neuroscience intensive care unit (NICU), and coronavirus disease 2019 intensive care unit (COVID-19 ICU). We found that pMRI was able to capture ischemic infarcts throughout the whole brain and detect lesions as small as 4 mm in diameter. We also report our experience of using pMRI to provide first-line diagnostic imaging and serial neurological monitoring in critically ill patients with ischemic stroke. Ischemic infarct volume measurements were consistent between low-field pMRI and conventional high-field MRI exams. Moreover, low-field pMRI stroke volumes significantly correlated with stroke severity at the time of exam and functional outcome at discharge. These findings validate the use of low-field pMRI as a novel imaging solution for patients with ischemic stroke.

## RESULTS

### Patient characteristics and imaging protocol

Low-field pMRI was used to obtain bedside intracranial imaging for 50 patients with ischemic stroke {23 female (46%); median [interquartile range (IQR)] age, 61 [55 to 71] years} (Table 1). Using pMRI, six patients were imaged at two serial time points, and one patient was imaged at three serial time points. A total of 58 low-field pMRI exams were obtained at an average of 37 ± 60 (SD) hours after the patient’s last known normal time (unknown for five patients). Specifically, 16 pMRI exams were obtained in the acute phase (≤24 hours), 33 in the subacute phase (24 hours to 7 days), and 4 in the chronic phase (>1 week).

**Table 1.** Patient demographics and clinical characteristics. y, year; no., number; NIHSS, National Institutes of Health Stroke Scale; LKN, last known normal; hr., hour.

**Characteristics**	**Patient cohort (*n* = 50)**
Age, median (IQR), y	61 (55–71)
Female, no. (%)	23 (46)
Race, no. (%)	
White	33 (66)
Black/African American	8 (16)
Asian	3 (6)
Other/not listed	6 (12)
Baseline medical history, no. (%)	
Atrial fibrillation	5 (10)
Coronary artery disease	6 (12)
Diabetes mellitus	10 (20)
Hypertension	34 (68)
Hyperlipidemia	24 (48)
Prior stroke	4 (8)
NIHSS at admission, median (IQR)*	5 (3–8)
NIHSS at pMRI exam, median (IQR)^†^	4 (1–11)
Time from LKN to pMRI exam, median (IQR), hr^‡^	37 (19–82)
Discharge modified Rankin scale score, median (IQR)	3 (1–4)

*NIHSS values at admission were unavailable for four patients.

†NIHSS values at pMRI exam were unavailable for five patients.

‡LKN was unknown for five patients.

Our pMRI imaging protocol included T2W, FLAIR, and DWI [with apparent diffusion coefficient (ADC) maps] sequences. The mean examination time was 25:13 ± 1:16 minutes:seconds (min:s) (T2W, 7:06 ± 0:05; FLAIR, 11:07 ± 1:27; and DWI, 6:59 ± 1:13 min:s). A total of 50 T2W, 51 FLAIR, and 56 DWI images were obtained with pMRI. One T2W, one FLAIR, and six DWI images were substantially degraded because of patient motion and were excluded from analyses. All pMRI exams were performed in environments that contained ferromagnetic material, such as standard monitoring devices, infusion pumps, mechanical ventilators, and hemodialysis machines. The low magnetic field of the pMRI did not compromise the functionality of nearby hospital equipment. The pMRI operator and nurse were able to freely enter and exit the patient’s room without projectile risk. No adverse events or complications occurred.

### pMRI findings

**Fig. 1.**
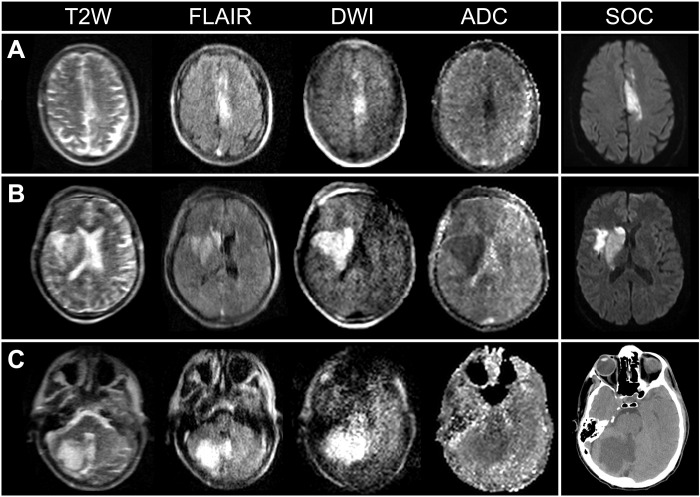
pMRI capturing ischemic infarcts throughout multiple regions of brain. (**A**) A 65-year-old male with a left anterior cerebral artery stroke imaged by pMRI and standard-of-care (SOC) MRI (1.5 T) (32 and 29 hours since last known normal, respectively). (**B**) A 61-year-old male with a right middle cerebral artery stroke imaged by pMRI and SOC MRI (1.5 T) (29 and 12 hours since last known normal, respectively). (**C**) A 57-year-old male with a right cerebellar stroke imaged by pMRI and SOC computed tomography (44 and 32 hours since last known normal, respectively). SOC exams included MRI (DWI sequence) and computed tomography.

**Fig. 2.**
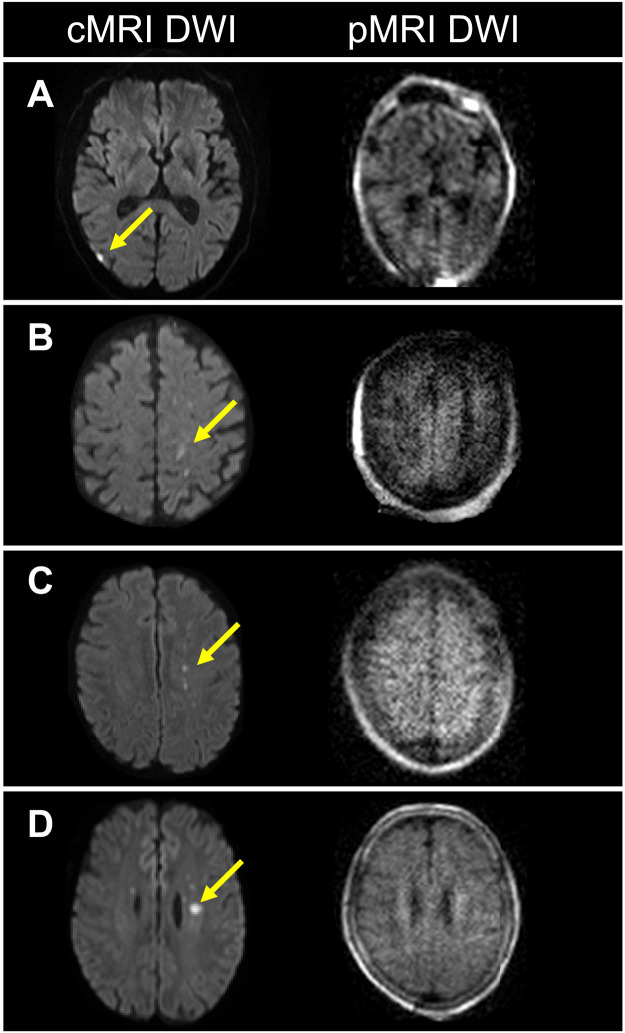
Patients with ischemic stroke with infarcts not visualized by pMRI. Four patients with ischemic stroke (A to D) had foci of restricted diffusion (yellow arrows) that were detected solely by conventional MRI (cMRI) (1.5/3 T) DWI sequences, but not pMRI DWI. (**A**) 7-mm-diameter infarct. (**B**) 10-mm infarct. (**C**) 4-mm infarct. (**D**) 7-mm infarct.

Each of the 50 patients with ischemic stroke examined by pMRI had an ischemic infarct detected by a standard-of-care neuroimaging exam (NCCT or 1.5/3-T MRI) obtained within 36 hours of the pMRI exam. Low-field (0.064 T) pMRI exams recapitulated ischemic infarcts in 45 (90%) of these patients with a sequence-specific sensitivity of 98% for T2W, 100% for FLAIR, and 86% for DWI (Fig. 1). The five patients in whom infarcts were not detected had small foci of restricted diffusion (range, 4 to 10 mm in diameter) that were captured only by high-field MRI DWI sequences. The corresponding pMRI DWI image for one patient was degraded by motion and uninterpretable; the pMRI DWI images for the other four patients were interpretable but failed to demonstrate the lesion (Fig. 2).

**Fig. 3.**
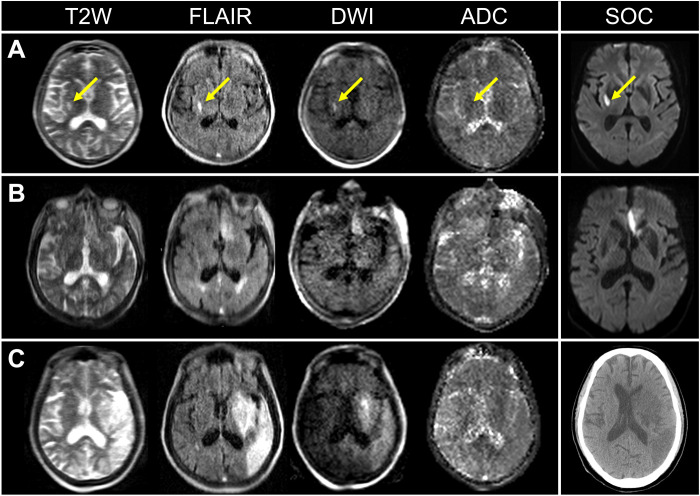
Ischemic infarcts of various lesion sizes shown on pMRI. (**A**) A 75-year-old female with a right middle cerebral artery infarct (13 mm in diameter) (yellow arrows) imaged by pMRI and SOC MRI (3 T; FLAIR) (30 and 48 hours since last known normal, respectively). (**B**) A 91-year-old female who received mechanical thrombectomy for the removal of a left anterior cerebral artery thrombus at 3 hours since last known normal. Subsequent pMRI and SOC MRI exams 13 and 19 hours after the neurointervention, respectively, detected a left anterior cerebral artery infarct (32 mm in diameter). (**C**) A 69-year-old male with a left middle cerebral artery infarct (114 mm in diameter) imaged by pMRI and SOC computed tomography (114 and 104 hours since last known normal, respectively). SOC exams included MRI (DWI sequence) and computed tomography.

Low-field pMRI exams demonstrated ischemic lesions as demarcated areas of hyperintensity on T2W, FLAIR, and DWI sequences and regions of reduced ADC values (Fig. 1). Point-of-care pMRI captured ischemic lesions in all brain regions, including those supplied by anterior cerebral (*n* = 2), middle cerebral (*n* = 35), posterior cerebral (*n* = 3), and cerebellar arteries (*n* = 3). Infarcts in watershed zones were also detected (*n* = 2). Ischemic infarcts over a range of lesion sizes were visualized by pMRI (range, 4 to 150 mm) (Fig. 3).

Low-field pMRI was deployed in the ED (*n* = 6), NICU (*n* = 40), and COVID-19 ICU (*n* = 4). In the COVID-19 ICU, low-field pMRI enabled neuroimaging that was otherwise unavailable or challenging to obtain because of patient clinical instability and infection control concerns during the beginning of the COVID-19 pandemic. For two (50%) of the COVID-19 patients, pMRI detected an otherwise unknown ischemic stroke, serving as a first-line diagnostic tool that prompted subsequent conventional neuroimaging studies, which were confirmatory, and guided clinical treatment.

**Fig. 4.**
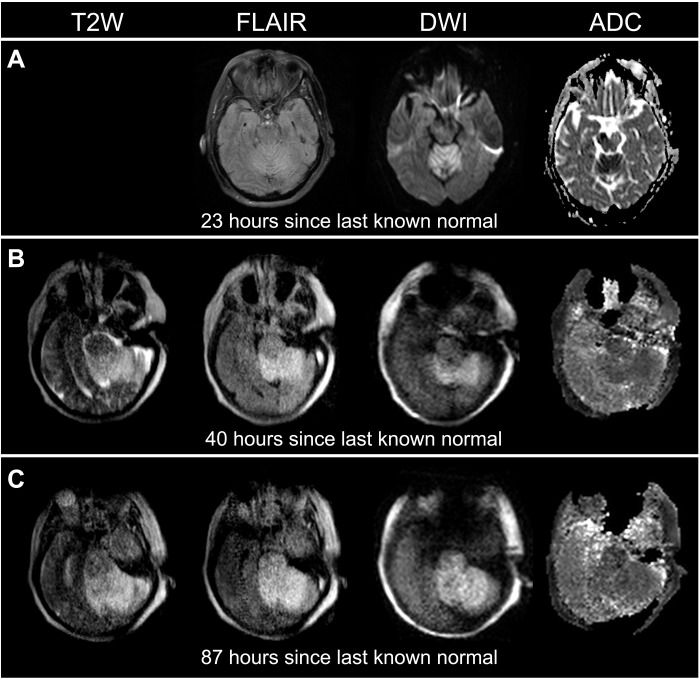
Serial imaging with pMRI facilitated dynamic monitoring of acute ischemic stroke progression in a 66-year-old female with a brainstem stroke. (**A**) The SOC MRI (3 T) was obtained 23 hours since last known normal. (**B**) The first pMRI exam was obtained 40 hours since last known normal. (**C**) The second pMRI exam was obtained 87 hours since last known normal. Serial pMRI exams (B and C) revealed an evolution and extension of cerebral infarction from the cerebellum into the brainstem (70.430 to 97.681 cm^3^). The SOC MRI exam did not include a T2W sequence.

**Fig. 5.**
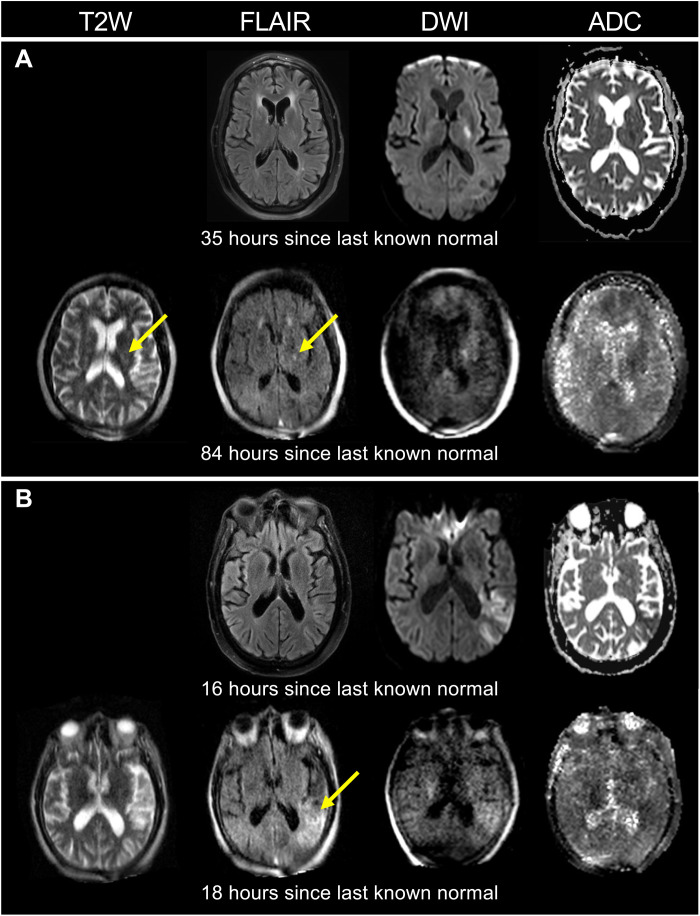
Monitoring of ischemic stroke development with pMRI. (**A**) A 66-year-old male with a left internal capsule stroke (4-mm infarct). A pMRI exam 49 hours after the initial SOC MRI (3 T) exam demonstrated the interval development of hyperintensity on the T2W and FLAIR sequences (yellow arrows). (**B**) A 90-year-old male with a left middle cerebral artery stroke. A pMRI exam 2 hours after the initial SOC MRI (3 T) exam revealed the interval development of hyperintensity on the FLAIR sequence (yellow arrow). The SOC MRI exams did not include the T2W sequence.

In addition, pMRI enabled neurological monitoring over a dynamic time profile for patients with ischemic stroke. Figure 4 illustrates a patient with a brainstem stroke who received pMRI imaging at two serial time points. The pMRI exams demonstrated an evolution and extension of acute ischemic infarction from the cerebellum into the brainstem. Figure 3B illustrates a pMRI exam obtained for a patient 13 hours after undergoing mechanical thrombectomy for the removal of a left anterior cerebral artery embolus. The pMRI exam detected acute ischemia after the neurointervention, and a conventional high-field MRI exam later confirmed this neuroimaging finding. Figure 5 shows two patients who received high-field MRI exams that demonstrated restricted diffusion on the DWI sequence but did not reveal hyperintensities on the conventional T2W or FLAIR sequences. Subsequent low-field pMRI exams recapitulated restricted diffusion on DWI sequences and demonstrated the interval development of hyperintensity on T2W and FLAIR sequences.

### Stroke volumetric analyses

Two raters (M.M.Y. and A.M.P.) manually measured infarct volumes on T2W, FLAIR, and DWI sequences of each pMRI exam and, if available, a conventional MRI (cMRI) exam acquired within 36 hours of the pMRI exam. Of the 58 pMRI exams obtained in this study, 37 had a cMRI exam within this time frame. Specifically, 16 pMRI exams were obtained before the corresponding cMRI exam (median [IQR] time between exams, 13 [4 to 21] hours), and 21 pMRI exams were obtained after the corresponding cMRI exam (median [IQR] time between exams, 16 [7 to 22] hours).

Intraclass correlation coefficient (ICC) analyses demonstrated significant interrater agreement for pMRI {ICC = 0.990, 95% confidence interval (CI): [0.985 to 0.993], *P* < 0.001} and cMRI volume measurements (ICC = 0.996, 95% CI: [0.993 to 0.997], *P* < 0.001). Stroke volume measurements were averaged between the two raters for T2W, FLAIR, and DWI images. Manually segmented infarcts on pMRI T2W, FLAIR, and DWI sequences had a median [IQR] infarct volume of 16.15 cm^3^ [5.01 to 97.68], 18.68 cm^3^ [4.50 to 89.65], and 23.31 cm^3^ [6.32 to 91.88], respectively.

**Fig. 6.**
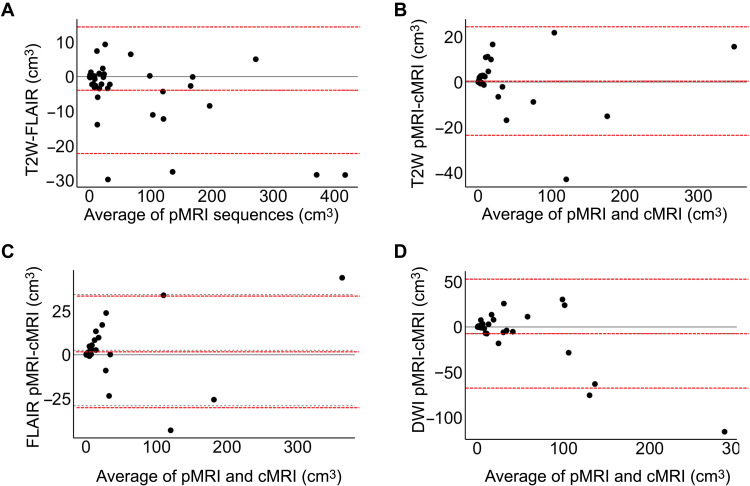
Bland-Altman plots of stroke volume measurements. Bland-Altman plots are shown for (**A**) pMRI T2W and FLAIR sequences {bias of −3.92 cm^3^ [limits of agreement (LOA): −22.26 to 14.43 cm^3^]}, (**B**) T2W pMRI and cMRI sequences [bias of 0.50 cm^3^ (LOA: −23.46 to 24.46 cm^3^)], (**C**) FLAIR pMRI and cMRI sequences [bias of 2.61 cm^3^ (LOA: −29.18 to 34.40 cm^3^)], and (**D**) DWI pMRI and cMRI sequences [bias of −7.15 cm^3^ (LOA: −67.59 to 53.29 cm^3^)]. Bias and LOA are shown by the dotted lines.

The consistency of stroke volume measurements across pMRI structural sequences (T2W and FLAIR) was evaluated using ICC and Bland-Altman analyses. There was significant agreement between pMRI T2W and FLAIR volume measurements (ICC = 0.997, 95% CI: [0.995 to 0.999], *P* < 0.001). The Bland-Altman plot of T2W and FLAIR volume measurements (T2W-FLAIR stroke volumes) showed a bias of −3.92 cm^3^ and limits of agreement from −22.26 to 14.43 cm^3^ (Fig. 6A).

Stroke volume measurements obtained from pMRI were validated against those obtained from cMRI. Low-field pMRI infarct volumes strongly correlated with cMRI volumes for T2W (ICC = 0.994, 95% CI: [0.986 to 0.997], *P* < 0.001), FLAIR (ICC = 0.989, 95% CI: [0.976 to 0.995], *P* < 0.001), and DWI (ICC = 0.940, 95% CI: [0.871 to 0.972], *P* < 0.001) sequences. The Bland-Altman plots (pMRI-cMRI stroke volumes) showed a bias of 0.50 cm^3^ with limits of agreement from −23.46 to 24.46 cm^3^ for T2W sequences (Fig. 6B), a bias of 2.61 cm^3^ with limits of agreement from −29.18 to 34.40 cm^3^ for FLAIR sequences (Fig. 6C), and a bias of −7.15 cm^3^ with limits of agreement from −67.59 to 53.29 cm^3^ (Fig. 6D).

**Fig. 7.**
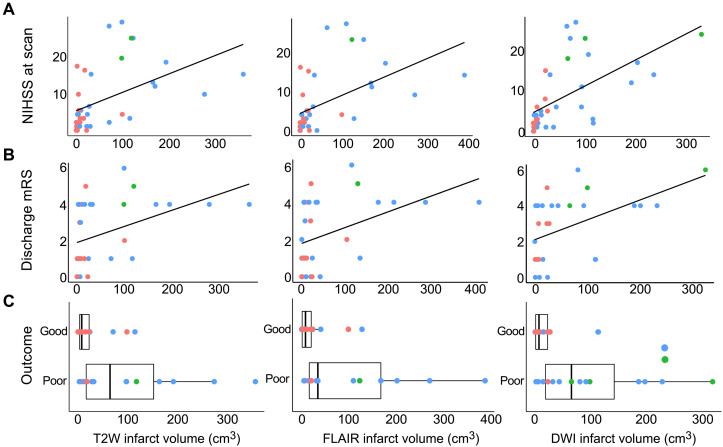
Stroke volume and clinical status. Low-field pMRI infarct volume measurements predicted (**A**) stroke severity at the time of scan [Spearman correlation (*r*_s_); T2W: *r*_s_ = 0.583, *P* < 0.001; FLAIR: *r*_s_ = 0.623, *P* < 0.001; DWI: *r*_s_ = 0.696, *P* < 0.001] and (**B**) mRS scores at discharge (T2W: *r*_s_ = 0.487, *P* < 0.01; FLAIR: *r*_s_ = 0.465, *P* < 0.01; DWI: *r*_s_ = 0.503, *P* < 0.01). (**C**) Boxplots illustrating stroke volumes for patients with good functional outcome (mRS: 0 to 3) and poor functional outcome (mRS: 4 to 6). Compared to patients with good functional outcome, those with poor functional outcomes had larger infarct volumes measured on pMRI T2W (*P* < 0.01), FLAIR (*P* < 0.01), and DWI (*P* < 0.01) sequences. Phase of stroke is shown in color (red, acute; blue, subacute; green, chronic).

Last, the relationship between pMRI stroke volume and clinical status was evaluated. Infarct volumes significantly correlated with stroke severity [National Institutes of Health Stroke Scale (NIHSS)] at the time of the pMRI scan (T2W: *r*_s_ = 0.583, *P* < 0.001; FLAIR: *r*_s_ = 0.623, *P* < 0.001; and DWI: *r*_s_ = 0.696, *P* < 0.001) (Fig. 7A) and functional outcome [modified Rankin scale (mRS)] at discharge (T2W: *r*_s_ = 0.487, *P* < 0.01; FLAIR: *r*_s_ = 0.465, *P* < 0.01; and DWI: *r*_s_ = 0.503, *P* < 0.01) (Fig. 7B). Compared to patients with good functional outcome (mRS: 0 to 3), those with poor functional outcome (mRS: 4 to 6) had larger infarct volumes measured on T2W (median [IQR] infarct volume, good: 7.74 [2.85 to 22.74] cm^3^; poor: 64.56 [14.68 to 170.63] cm^3^, *P* < 0.01), FLAIR (median [IQR] infarct volume, good: 8.34 [1.10 to 21.52] cm^3^; poor: 34.23 [15.75 to 183.24] cm^3^, *P* < 0.01), and DWI (median [IQR] infarct volume, good: 7.31 [1.30 to 23.45] cm^3^; poor: 65.56 [14.50 to 186.01] cm^3^, *P* < 0.01) sequences (Fig. 7C).

Binary logistic regression analyses were also performed to evaluate the relationship between pMRI stroke volume and discharge functional outcome (good or poor). In the unadjusted model, infarct volumes were significantly associated with functional outcome for T2W {common odds ratio (cOR) = 1.018, 95% CI: [1.005 to 1.039], *P* < 0.05}, FLAIR (cOR = 1.019, 95% CI: [1.006 to 1.041], *P* < 0.05), and DWI (cOR = 1.020, 95% CI: [1.005 to 1.043], *P* < 0.05) sequences. In the multivariable model adjusted for baseline prognostic variables, infarct volumes were significantly associated with functional outcome for T2W {adjusted cOR (acOR) = 1.032, 95% CI: [1.009 to 1.070], *P* < 0.05} and FLAIR (acOR = 1.039, 95% CI: [1.010 to 1.085], *P* < 0.05) sequences, but not DWI (acOR = 1.018, 95% CI: [0.999 to 1.051], *P* = 0.128) (Table 2).

**Table 2.** pMRI stroke volumes and functional outcome at discharge.

**pMRI** **sequence**	**cOR (95% CI)**	***P* value**	acOR (95% CI)*	***P* Value**
T2W	1.018 (1.005–1.039)	0.029	1.032 (1.009–1.070)	0.032
FLAIR	1.019 (1.006–1.041)	0.026	1.039 (1.010–1.085)	0.030
DWI	1.020 (1.005–1.043)	0.034	1.018 (0.999–1.051)	0.128

*Multivariable binary logistic regression model, adjusting for age, sex, race, medical intervention (intravenous thrombolytics and mechanical thrombectomy), NIHSS at admission, diabetes mellitus, and atrial fibrillation.

## DISCUSSION

We deployed a highly mobile 0.064-T pMRI scanner into inpatient and emergency care settings and accurately demonstrated and characterized ischemic stroke at the bedside. Consistent with high-field cMRI, ischemic infarcts appeared as demarcated regions of hyperintensity on T2W, FLAIR, and DWI images and as dark regions on ADC maps. Ischemic lesions over a range of lesion sizes were detected in cortical, subcortical, and cerebellar regions of the brain. Stroke volume measurements were consistent across pMRI structural sequences, and pMRI stroke volume measurements were in agreement with cMRI measurements. Low-field pMRI stroke volume measurements significantly correlated with stroke severity at the time of exam and functional outcome at discharge, recapitulating a well-established clinical relationship (*27*–*32*). These results extend earlier findings (*22*, *24*) while validating the use of pMRI to obtain clinically valuable neuroimaging for patients with ischemic stroke.

Brain imaging is crucial to the clinical management of patients with ischemic stroke. However, neuroimaging of critically ill patients with stroke can be challenging because transport of patients to dedicated radiologic suites is associated with numerous risks (*7*–*14*). We demonstrate that pMRI can provide clinically useful neuroimaging when the disadvantages of intrahospital transport are prohibitive. For example, two intensive care COVID-19 patients presenting with neurological deficits were imaged by pMRI at the bedside when transport to conventional NCCT or cMRI was initially deemed clinically unfeasible. Low-field pMRI enabled the detection of otherwise unknown ischemic strokes for these patients. The positive findings by bedside pMRI prompted the clinical team to acquire confirmatory neuroimaging studies, which enabled the providers to appropriately adjust the patients’ targeted treatment plans. These findings indicate that low-field pMRI can serve as a valuable imaging approach in intensive care settings.

Bedside pMRI can also provide clinically useful neuroimaging throughout the clinical course of patients with ischemic stroke. Patients with stroke often require frequent neuroimaging to monitor the progression of the stroke and assess the efficacy of treatments (*4*, *33*). Repeated transport of critically ill patients for conventional neuroimaging studies may be unfeasible because of the risks of intrahospital transport (*7*–*14*). Low-field pMRI may serve as a valuable solution to serial neuroimaging needs, as consecutive, serial imaging with pMRI was demonstrated to be feasible in this study. For one patient, two serial pMRI exams obtained 37 hours apart from each other were able to track the evolution of the patient’s brainstem stroke. In addition, pMRI provided clinically useful follow-up imaging to conventional neurological exams for two patients, demonstrating the interval development of hyperintensity on T2W and FLAIR sequences and confirming the presence of irreversible cerebral ischemia. Last, pMRI was used in the postinterventional setting, detecting the progression of ischemic stroke for one patient who received mechanical thrombectomy. These results demonstrate that pMRI can facilitate serial and postoperative imaging throughout an extended time course.

Low-field pMRI may also facilitate the use of MRI in the emergency care setting. Upon arrival to the ED, patients with a suspected diagnosis of stroke typically receive a NCCT exam to exclude the presence of blood before administering intravenous thrombolytics (*34*, *35*). However, high-field MRI is superior to NCCT because it can detect intracerebral hemorrhage with equal accuracy (*6*, *36*, *37*), avoids risks from ionizing radiation (*18*–*21*), and is uniquely capable of diagnosing acute ischemia with DWI (*6*, *38*, *39*). Despite the clinical strengths of high-field MRI, it is often inaccessible in acute stroke care because of the logistical barriers of transporting patients to strictly controlled high-field MRI suites (*40*). Low-field pMRI can potentially offer an avenue to access the benefits of MRI in the emergency care setting because pMRI can be deployed directly to the ED. In this study, low-field pMRI was used to image six patients with ischemic stroke upon their immediate arrival to the ED, detecting ischemic infarcts in four (66%) patients; the other two had small watershed infarcts undetected by low-field pMRI. In another recent report, low-field pMRI was found to detect intracerebral hemorrhage with clinically significant accuracy, and five of the patients included in the report were scanned in the ED (*24*). These preliminary findings demonstrate the feasibility and use of low-field pMRI to detect ischemic stroke and blood in the ED. This novel imaging approach has the potential to expedite stroke diagnosis and treatment early in the continuum of stroke care, although further experience and study of low-field pMRI in the emergency care setting are necessary.

In addition, low-field pMRI can facilitate access to MRI in resource-limited settings. High-field MRI scanners (1.5 to 3 T) are expensive to purchase (~$1 million/T) (*40*). These superconducting magnets also require costly infrastructure and highly trained MRI technicians for operation (*40*). These financial barriers have restricted the availability of MRI scanners in both state-of-the-art hospitals (*15*) and under-resourced facilities and developing areas (*26*). In contrast to high-field MRI, low-field MRI technologies are a substantially more affordable imaging modality. In contrast to high-field MRI, low-field MRI technologies are a significantly more affordable imaging modality. This relates to the reduced cost of manufacturing, maintenance and operation (*41*). The low magnetic field strength of the pMRI also enables the device to be operated in environments containing ferromagnetic material and integrates electromagnetic interference rejection (*42*), removing the need for cost-prohibitive MRI suites. Moreover, the use of low-field pMRI requires minimal training of providers and therefore precludes the need for specialized technicians (*23*). For these reasons, low-field pMRI can both supplement hospitals that currently have limited MRI scanners and provide otherwise unavailable imaging in community-based, low-income, and rural health centers.

Despite the clinical potential of pMRI, continued improvements in pMRI imaging quality is required, most notably for the DWI sequence. For both high-field cMRI and low-field pMRI, the DWI sequence inherently has a lower signal-to-noise ratio (SNR) compared to other imaging sequences (T2W and FLAIR) due to its long diffusion preparation gradients. This limitation is further amplified by the reduced SNR associated with the use of a low magnetic field (*40*). Consequently, pMRI failed to detect small foci of restricted diffusion for five patients in this study. In addition, the earliest version of the pMRI DWI sequence (software version RC6; *n* = 12 patients) was acquired with a three-dimensional (3D) diffusion-weighted steady-state free precession, while the later versions (software versions RC7 and RC8; *n* = 38 patients) were turbo spin echo sequences. This change in sequence parameters may have limited our ability to assess the sensitivity of pMRI DWI. Therefore, further study and optimization of the pMRI DWI is necessary. In parallel, the development and implementation of imaging techniques—such as deep learning–based image sequences, automated diagnosis algorithms (*43*), and advanced image reconstruction approaches [domain-transform manifold learning (*44*)]—may further support the diagnostic capabilities of pMRI.

Continued use of pMRI to image patients with ischemic stroke over an extended time course is also warranted. In this study, most of the patients were imaged in the subacute phase of stroke. Because the appearance of stroke changes over time (*45*–*49*), continued study of stroke patients in the hyperacute, acute, and chronic phases of stroke is necessary to establish a complete temporal profile of ischemia on low-field pMRI. Notably, continued use of pMRI in the ED setting will facilitate future study of pMRI in evaluating ischemic stroke in the hyperacute and acute setting. Another limitation of the study is that the pMRI exams were not assessed by blinded reviewers in this study. When evaluating the presence of an infarct on pMRI images, each pMRI exam was compared to the standard-of-care imaging study acquired closest to the time of the pMRI exam as a reference point. This approach may have introduced bias, although the current study provides valuable insight into the ability of pMRI to demonstrate ischemic stroke. Therefore, future blind-rater analyses are necessary to further delineate the diagnostic capabilities of low-field pMRI for ischemic stroke across all phases of stroke.

It is also important to note that patients with cardiovascular implantable devices were not included in this study or prior reports. However, a growing body of evidence has shown high-field 1.5-T MRI to be feasible for patients with cardiovascular implants (*50*–*53*). Therefore, low–magnetic field pMRI (0.064 T) should be compatible with this patient population, although further study and replication of prior results is necessary.

The 0.064-T pMRI scanner used in this study operates at very low magnetic field strength (<0.1 T). Although MRI systems operating at higher magnetic field strength [e.g., mid-field (0.3 to 1.0 T)] have increased SNR and spatial resolution, there are unique advantages to the use of very low-field MRI. For example, T1 dispersion is known to be increased at very low magnetic field strength (*54*), which positions low-field MRI as a promising tool for developing endogenous contrasts. Moreover, low-field MRI scanners have fewer shielding, cooling, and weight constraints relative to higher field systems, enabling low-field scanners to be portable. The clinical benefit provided by portable imaging approaches requires further investigation. However, recent evidence suggests that bringing imaging directly to patients with stroke may facilitate therapeutic decision-making and improve clinical outcome (*55*). Our work now moves the hypothesis testing of whether pMRI imaging adds value to stroke care from a theoretical notion to one that can be tested empirically. Point-of-care technologies, such as ultrasound, have changed how clinical teams acquire, interpret, and act on available patient data, and these results lay the groundwork for similar advancements in MRI.

In conclusion, we deployed low-field pMRI to detect ischemic stroke at the bedside. This approach enabled otherwise unavailable neuroimaging for select critically ill patients, serial and postoperative imaging throughout the clinical course of patients, and acute stroke imaging in the emergency care setting. These results demonstrate that low-field pMRI can mitigate risks associated with transport to conventional neuroimaging, facilitate the evaluation of stroke over a dynamic clinical course, and potentially enable earlier diagnosis and treatment of ischemic stroke. Moreover, the ease of use and low cost of pMRI position this novel imaging modality to address clinical bottlenecks and unmet imaging needs in resource-limited settings. Together, these results suggest that low-field pMRI can create imaging pathways that circumvent challenges and limitations associated with traditional stroke imaging approaches.

## METHODS

### Study design and participants

This prospective observational study was performed from July 2019 to December 2020 in the NICU, ED, and COVID-19 ICU of Yale New Haven Hospital. From July 2019 to March 2020, the study operated under a research protocol approved by Yale’s Institutional Review Board with an investigational device exemption. During this time, informed consent was obtained for all patients included in the study. From March 2020 to August 2020, pMRI exams were obtained as part of clinical care under the U.S. Food and Drug Administration (FDA) general clearance for portable imaging systems during the COVID-19 pandemic (*56*). On 11 August 2020, the FDA granted specific approval to the pMRI, and pMRI imaging studies continued to be obtained as part of the patient’s clinical care without research consent. All pMRI exams were performed in accordance with relevant guidelines and policies as informed by the Yale Human Research Protection Program and U.S. FDA.

Patients admitted to the NICU, ED, and COVID-19 ICU were screened for eligibility. Inclusion criteria for patients in the NICU entailed a standard-of-care NCCT or MRI exam indicating neuropathology, such as intracerebral hemorrhage or ischemic stroke. Inclusion criteria for patients in the ED entailed a suspected diagnosis of stroke (i.e., patients with a stroke alert code). In the COVID-19 ICU, inclusion criteria entailed a demonstrated or suspected neurological deficit as indicated by the clinical care team. For all patients, exclusion criteria included an inability to lay flat, patient body habitus exceeding the dimensions of the pMRI (see the “Technical and imaging parameters” section below), or the presence of MRI contraindications (e.g., cardiac pacemakers, insulin pumps, deep brain stimulators, and cochlear implants). In the current study, we evaluated only patients with a clinical diagnosis of ischemic stroke as confirmed by standard-of-care neuroimaging (NCCT or 1.5/3-T MRI). A subset of these patients was also studied in a prior report (*n* = 9) (*22*). Clinical data were collected from each patient’s electronic medical record.

### Technical and imaging parameters

The 0.064-T pMRI device (Hyperfine, Guilford, CT, USA) has a height of 140 cm and a width of 86 cm. The device uses an eight-channel radiofrequency head coil, which has a height of 26 cm and a width of 20 cm. The vertical and horizontal clearance of the pMRI are 32 and 55 cm, respectively. The biplanar three-axis gradient system has a peak gradient amplitude of 26 mT/m (on *z* axis) and 25 mT/m (on *x* and *y* axes). The gradient amplifiers give a maximum of 60-A current and a gradient field of 25 mT/m for each axis. The pMRI system operates from a standard 110 V, 15-A electrical outlet and does not require any liquid cryogens. The device integrates electromagnetic interference rejection (*42*), removing the need for a shielded room.

All pMRI exams were conducted at the bedside of patients in single-patient ICU rooms or ED care rooms. The scanning environment included the presence of standard hospital equipment, including vital signs monitors (e.g., cardiac telemetry and pulse oximetry), intravenous infusion pumps, ventilators, compressed gas cylinders, and dialysis machines. The pMRI was operated by clinical research staff experienced in performing pMRI exams. During the acquisition of pMRI exams, health care providers freely entered and exited the patient’s room without any projectile risk. The open geometry design of the pMRI enabled nurses to access the patient for medical treatment (e.g., drug infusions, blood glucose monitoring, and temperature recording) during scan acquisition.

The imaging protocol for patients with ischemic stroke included 3D T2W, FLAIR, and DWI sequences. ADC maps were generated from the DWI sequences. These sequences underwent multiple software updates (RC6, *n* = 12; RC7, *n* = 2; and RC8, *n* = 34) throughout the study. Throughout these updates, T2W parameters included (RC6/RC7/RC8) were as follows: acquisition time = 7:09/7:10/7:01 min:s, repetition time (TR) = 2200/2200/2200 ms, echo time (TE) = 234/253/253 ms, 1.5 mm by 1.5 mm by 5 mm/1.5 mm by 1.5 mm by 5 mm/1.5 mm by 1.5 mm by 5 mm resolution, 36/36/36 slices, and slice thickness of 5/5/5 mm. The FLAIR parameters included were as follows: acquisition time = 11:24/12:25/9:33 min:s, TR = 4000/4000/4000 ms, TE = 238/253/228 ms, 1.5 mm by 1.5 mm by 5 mm/1.5 mm by 1.5 mm by 5 mm/1.6 mm by 1.6 mm by 5 mm resolution, 36/36/36 slices, and slice thickness of 5/5/5 mm. The DWI parameters included were as follows: acquisition time = 8:03/5:25/7:31 min:s, TR = 33/1000/750 ms, TE = 17/99/96 ms, *b* = 830/1000/800 s/mm^2^, 3 mm by 3 mm by 5 mm/2 mm by 2 mm by 5 mm/2.4 mm by 2.4 mm by 6 mm resolution, 36/36/30 slices, and slice thickness of 5/5/6 mm. All sequences are turbo spin echo based, with the exception of the RC6 DWI sequence, which was acquired with a 3D diffusion-weighted steady-state free precession. All sequences were obtained in the axial plane with a field of view of 22 cm (anterior/posterior) by 18 cm (right/left) by 18 cm (inferior/superior) and number of averages = 1. Additional information on imaging parameters can be found in the Supplementary Materials.

Point-of-care pMRI exams were configured using an electronic tablet (iPad Pro third generation; Apple, Cupertino, CA, USA) that connected to the pMRI device’s local WiFi hotspot. Using the tablet, prescan calibrations, localizers, and sequences were arranged through a user interface hosted on a web browser. Images were displayed on the electronic tablet in real time throughout image acquisition and processing. Upon completion of the exam, pMRI images were automatically uploaded in DICOM format to a cloud-based server.

### Image analysis

Low-field pMRI was used to image patients with ischemic stroke at the bedside. Included in this analysis were patients whose standard-of-care imaging (NCCT or 1.5/3-T MRI) within 36 hours of the pMRI exam indicated the presence of an ischemic infarct. Patients with hemorrhagic transformation of the infarct during the time of the pMRI or standard-of-care imaging exam were not evaluated in this study because of the potential confounding effects of hemorrhagic transformation on subsequent volumetric, stroke severity, and functional outcome analyses. To evaluate the ability of pMRI to detect ischemic infarction, each low-field pMRI exam was evaluated and compared to the standard-of-care NCCT or MRI exam acquired closest to the time of the pMRI exam, which served as the gold standard. The standard-of-care imaging exam was first examined to confirm the presence of cerebral infarction and localize the lesion. The pMRI exam was then evaluated using the standard-of-care imaging exam as a reference point, and a pMRI exam was considered to have correctly captured an infarct if at least one sequence (T2W, FLAIR, or DWI) recapitulated the known infarct.

All pMRI exams that correctly demonstrated ischemic infarction were included in further volumetric analyses. These pMRI exams and the closest acquired cMRI exam within 36 hours of the pMRI exam, if available, were manually segmented by two low-field MRI core laboratory members (M.M.Y. and A.M.P.). These two raters used Horos (v4.0.0) to segment lesions on all available T2W, FLAIR, and DWI sequences for both pMRI and cMRI exams. Specifically, each rater traced a region of interest on each slice demonstrating an infarct. To calculate the total infarct volume of each imaging exam, each region of interest’s area was multiplied by the slice thickness, and the resultant values were summed across the full extent of the ischemic lesion. The individual infarct volume measurements for each rater were averaged together for each imaging exam. Each rater segmented all pMRI images before cMRI images and was blinded to clinical data and patient identifiers during the segmentation of the images.

### Statistical analysis

Descriptive statistics are presented as mean (SD) or median (IQR) as appropriate. Interrater agreement was assessed by computing ICCs between the rater’s infarct volume assessments.

Consistency of stroke volumes across pMRI structural sequences was assessed by computing the ICC between average T2W and FLAIR stroke volume measurements. Intersequence agreement was also studied by the Bland-Altman method with calculation of bias and limits of agreement. After assessing the consistency of stroke volumes across pMRI sequences, pMRI stroke volume measurements were validated against those obtained from cMRI images. To this end, ICC and Bland-Altman analyses were performed between pMRI and cMRI volume measurements for T2W, FLAIR, and DWI sequences.

In addition to volumetric analyses, the relationship between low-field pMRI stroke volume and clinical status was evaluated. For T2W, FLAIR, and DWI sequences, Spearman correlation analyses were performed between infarct volume and stroke severity (NIHSS) at the time of the pMRI scan. Similarly, Spearman correlation analyses were performed between infarct volume measurement and functional outcome (mRS) at discharge.

Binary logistic regression analyses were also performed to assess the relationship between pMRI stroke volume and discharge functional outcome. mRS scores were dichotomized into good (0 to 3) and poor (4 to 6) outcomes (*57*). The effect of pMRI stroke volume on functional outcome was expressed as unadjusted COR and acOR. To adjust for baseline prognostic variables, the adjusted models included age, sex, race, medical intervention (intravenous thrombolytics, mechanical thrombectomy, or both interventions), NIHSS at admission, diabetes mellitus, and atrial fibrillation. Wilcoxon rank sum tests were also performed to compare stroke volume between patients with good and poor functional outcome.

Patients with COVID-19 were not included in these correlation or regression analyses because of the confounding effect of COVID-19 on outcome. All statistical analyses were performed using RStudio version 1.2.5033.

## Supplementary Materials

### This PDF file includes:

Imaging Parameters Table S1 References

## Notes

View/request a protocol for this paper from *Bio-protocol*.
