# Supplementary material for: Portable, low-field magnetic resonance imaging enables highly accessible and dynamic bedside evaluation of ischemic stroke
Source: Sci Adv. 2022 Apr 20;8(16):eabm3952. doi: 10.1126/sciadv.abm3952 (PMC9020661; doi:10.1126/sciadv.abm3952)
Supplement: Supplementary file 1 — Imaging Parameters Table S1 References [file sciadv.abm3952_sm.pdf]

Science Advances
AAAS

# Supplementary Materials for

## Portable, low-field magnetic resonance imaging enables highly accessible and dynamic bedside evaluation of ischemic stroke

Matthew M. Yuen, Anjali M. Prabhat, Mercy H. Mazurek, Isha R. Chavva, Anna Crawford, Bradley A. Cahn, Rachel Beekman, Jennifer A. Kim, Kevin T. Gobeske, Nils H. Petersen, Guido J. Falcone, Emily J. Gilmore, David Y. Hwang, Adam S. Jasne, Hardik Amin, Richa Sharma, Charles Matouk, Adrienne Ward, Joseph Schindler, Lauren Sansing, Adam de Havenon, Ani Aydin, Charles Wira, Gordon Sze, Matthew S. Rosen, W. Taylor Kimberly*, Kevin N. Sheth*

*Corresponding author. Email: wtkimberly@mgh.harvard.edu (W.T.K.); kevin.sheth@yale.edu (K.N.S.)

Published 20 April 2022, *Sci. Adv.* **8**, eabm3952 (2022)
DOI: 10.1126/sciadv.abm3952

**This PDF file includes:**

Imaging Parameters
Table S1
References

## IMAGING PARAMETERS

The T2W and FLAIR sequences use a linear sample ordering that passes twice through the center of the k-space pattern at 25% and 75% of the way through each echo train to reduce blurring from T2W relaxation. The DWI sequence uses a radial-out sample ordering. The refocusing trains for all sequences use 180 degree, 150 μsec hard pulses, with quadratic phase cycling (*58, 59*). These sequence parameters and standard reconstruction and post-processing parameters are pre-set by the manufacturer (**Supplementary Table 1**).

The variable density k-space was gridded along two dimensions using a non-uniform fast Fourier Transform with sample density compensation. A uniform Fourier transform was used along the readout dimension. Image post-processing consisted of receive coil combination (*60*), receive coil intensity correction using a pre-set coil shading model, and geometric distortion correction for main B0 field inhomogeneity and gradient nonlinearity.

The DWI sequence underwent changes in parameters across software updates. The RC6 DWI sequence was acquired with a 3D diffusion-weighted steady state free precession (DW-SSFP) sequence (*61*). This sequence used a 14.4 mT/m diffusion gradient of length 25.2 msec, and 50 degrees flip angle. The b-value for this sequence was 849 s/mm$^2$ and was computed for T1 = 300 msec, and T2W = 90 msec, using DW-SSFP signal equations as described previously (*62*). RC7 and RC8 used a 3D turbo spin echo sequence preceded by a double spin echo DWI preparation section with two sets of bipolar gradients (*63, 64*). In RC8, each of the four bipolar gradients has an amplitude of 20 mT/m and 18 msec length, with a 4.7 msec gap between each gradient pulse. RC7 used the same gradients, but with a 2.7 msec gap. The time interval from the end of the 90 degree excitation to the center of the double spin echo DWI preparation segment is 44.7 and 40.7 msec for RC8 and RC7, respectively.

For all sequence versions, the DWI preparation pulses were applied only on the A/P direction gradient and had shapes optimized using the scanner's eddy current pre-emphasis model to utilize the maximum current limit. The b-values reported here for the diffusion preparation segments are the actual calculated b-values given these optimized gradient shapes and are [849, 820, and 880 s/mm$^2$] for [RC6, RC7, RC8].

**Supplementary Table 1. Additional Parameters for Portable MRI Sequences**

| | T2W | FLAIR | DWI |
|---|---|---|---|
| K-space sampling pattern | Elliptical, variable density cartesian, 20% undersampled. | Elliptical, variable density cartesian, 20% undersampled. | Variable density cartesian, 5x oversampled |
| Echo train sample ordering | Linear, two passes through k-space center | Linear, two passes through k-space center | Radial-out |
| Echo train length | 80 | 80 | 40 |
| Refocusing train type | 180 degree hard pulses, quadratic phase cycling | 180 degree hard pulses, quadratic phase cycling | 180 degree hard pulses, quadratic phase cycling |
| Inversion time / pulse type | N/A | 1.4 sec / adiabatic | N/A |

**REFERENCES AND NOTES**


1. W. Johnson, O. Onuma, M. Owolabi, S. Sachdev, Stroke: A global response is needed. *Bull. World Health Organ.* **94**, 634-634A (2016).

2. S. S. Virani, A. Alonso, H. J. Aparicio, E. J. Benjamin, M. S. Bittencourt, C. W. Callaway, A. P. Carson, A. M. Chamberlain, S. Cheng, F. N. Delling, M. S. V. Elkind, K. R. Evenson, J. F. Ferguson, D. K. Gupta, S. S. Khan, B. M. Kissela, K. L. Knutson, C. D. Lee, T. T. Lewis, J. Liu, M. S. Loop, P. L. Lutsey, J. Ma, J. Mackey, S. S. Martin, D. B. Matchar, M. E. Mussolino, S. D. Navaneethan, A. M. Perak, G. A. Roth, Z. Samad, G. M. Satou, E. B. Schroeder, S. H. Shah, C. M. Shay, A. Stokes, L. VanWagner, N. Y. Wang, C. W. Tsao; American Heart Association Council on Epidemiology and Prevention Statistics Committee and Stroke Statistics Subcommittee, Heart disease and stroke statistics-2021 update: A report From the American Heart Association. *Circulation* **143**, e254–e743 (2021).

3. V. L. Feigin, M. H. Forouzanfar, R. Krishnamurthi, G. A. Mensah, M. Connor, D. A. Bennett, A. E. Moran, R. L. Sacco, L. Anderson, T. Truelsen, M. O'Donnell, N. Venketasubramanian, S. Barker-Collo, C. M. Lawes, W. Wang, Y. Shinohara, E. Witt, M. Ezzati, M. Naghavi, C. Murray; Global Burden of Diseases, Injuries, and Risk Factors Study 2010 (GBD 2010) and the GBD Stroke Experts Group., Global and regional burden of stroke during 1990-2010: Findings from the Global Burden of Disease Study 2010. *Lancet* **383**, 245–255 (2014).

4. W. J. Powers, A. A. Rabinstein, T. Ackerson, O. M. Adeoye, N. C. Bambakidis, K. Becker, J. Biller, M. Brown, B. M. Demaerschalk, B. Hoh, E. C. Jauch, C. S. Kidwell, T. M. Leslie-Mazwi, B. Ovbiagele, P. A. Scott, K. N. Sheth, A. M. Southerland, D. V. Summers, D. L. Tirschwell, Guidelines for the early management of patients with acute ischemic stroke: 2019 update to the 2018 guidelines for the early management of acute ischemic stroke: A guideline for healthcare professionals from the American Heart Association/American Stroke Association. *Stroke* **50**, e344–e418 (2019).

5. C. Williamson, L. Morgan, J. P. Klein, Imaging in neurocritical care practice. *Semin. Respir. Crit. Care Med.* **38**, 840–852 (2017).

6. J. A. Chalela, C. S. Kidwell, L. M. Nentwich, M. Luby, J. A. Butman, A. M. Demchuk, M. D. Hill, N. Patronas, L. Latour, S. Warach, Magnetic resonance imaging and computed tomography in emergency

assessment of patients with suspected acute stroke: A prospective comparison. *Lancet* **369**, 293–298 (2007).

7. P. J. D. Andrews, I. R. Piper, N. M. Dearden, J. D. Miller, Secondary insults during intrahospital transport of head-injured patients. *Lancet* **335**, 327–330 (1990).

8. S. S. Braman, S. M. Dunn, C. A. Amico, R. P. Millman, Complications of intrahospital transport in critically ill patients. *Ann. Intern. Med.* **107**, 469–473 (1987).

9. I. Smith, S. Fleming, A. Cernaianu, Mishaps during transport from the intensive care unit. *Crit. Care Med.* **18**, 278–281 (1990).

10. J. O. Taylor, C. F. Landers, J. D. Chulay, W. B. Hood Jr., W. H. Abelmann, Monitoring high-risk cardiac patients during transportation in hospital. *Lancet* **2**, 1205–1208 (1970).

11. V. C. Veiga, N. F. Postalli, T. K. Alvarisa, P. P. Travassos, R. T. Da Silva Vale, C. Z. de Oliveira, S. S. O. Rojas, Adverse events during intrahospital transport of critically ill patients in a large hospital. *Rev. Bras. Ter. Intensiva.* **31**, 15–20 (2019).

12. E. Wallen, S. T. Venkataraman, M. J. Grosso, K. Kiene, R. A. Orr, Intrahospital transport of critically ill pediatric patients. *Crit. Care Med.* **23**, 1588–1595 (1995).

13. C. Waydhas, Intrahospital transport of critically ill patients. *Crit. Care* **3**, R83–R89 (1999).

14. F. M. P. Gimenez, W. H. B. de Camargo, A. C. B. Gomes, T. S. Nihei, M. W. M. Andrade, M. L. de A F Sé Valverde, L. D' Epiro de Souza Campos, D. C. Grion, J. Festti, C. M. C. Grion, Analysis of adverse events during intrahospital transportation of critically ill patients. *Crit. Care Res. Pract.* **2017**, 6847124 (2017).

15. M. Sarracanie, N. Salameh, Low-field MRI: How low can we go? A fresh view on an old debate. *Front. Phys.* **8**, 172 (2020).

16. Z. Rumboldt, W. Huda, J. W. All, Review of portable CT with assessment of a dedicated head CT scanner. *AJNR Am. J. Neuroradiol.* **30**, 1630–1636 (2009).

17. W. E. Butler, C. M. Piaggio, C. Constantinou, L. Niklason, R. G. Gonzalez, G. R. Cosgrove, N. T. Zervas, A mobile computed tomographic scanner with intraoperative and intensive care unit applications. *Neurosurgery* **42**, 1304–1310; discussion 1310-1301 (1998).

18. D. J. Brenner, H. Hricak, Radiation exposure from medical imaging: Time to regulate? *JAMA* **304**, 208–209 (2010).

19. H. Hricak, D. J. Brenner, S. J. Adelstein, D. P. Frush, E. J. Hall, R. W. Howell, C. H. McCollough, F. A. Mettler, M. S. Pearce, O. H. Suleiman, J. H. Thrall, L. K. Wagner, Managing radiation use in medical imaging: A multifaceted challenge. *Radiology* **258**, 889–905 (2011).

20. S. P. Power, F. Moloney, M. Twomey, K. James, O. J. O'Connor, M. M. Maher, Computed tomography and patient risk: Facts, perceptions and uncertainties. *World J. Radiol.* **8**, 902–915 (2016).

21. Z. Xie, X. Liao, Y. Kang, J. Zhang, L. Jia, Radiation exposure to staff in intensive care unit with portable CT scanner. *Biomed. Res. Int.* **2016**, 5656480 (2016).

22. K. N. Sheth, M. H. Mazurek, M. M. Yuen, B. A. Cahn, J. T. Shah, A. Ward, J. A. Kim, E. J. Gilmore, G. J. Falcone, N. Petersen, K. T. Gobeske, F. Kaddouh, D. Y. Hwang, J. Schindler, L. Sansing, C. Matouk, J. Rothberg, G. Sze, J. Siner, M. S. Rosen, S. Spudich, W. T. Kimberly, Assessment of brain injury using portable, low-field magnetic resonance imaging at the bedside of critically ill patients. *JAMA Neurol.* **78**, 41–47 (2021).

23. A. M. Prabhat, A. L. Crawford, M. H. Mazurek, M. M. Yuen, I. R. Chavva, A. Ward, W. V. Hofmann Jr, N. Timario, S. R. Qualls, J. Helland, C. Wira, G. Sze, M. S. Rosen, W. T. Kimberly, K. N. Sheth, Methodology for low-field, portable magnetic resonance neuroimaging at the bedside. *Front. Neurol.* **12**, 760321 (2021).

24. M. H. Mazurek, B. A. Cahn, M. M. Yuen, A. M. Prabhat, I. R. Chavva, J. T. Shah, A. L. Crawford, E. B. Welch, J. Rothberg, L. Sacolick, M. Poole, C. Wira, C. C. Matouk, A. Ward, N. Timario, A. Leasure, R. Beekman, T. J. Peng, J. Witsch, J. P. Antonios, G. J. Falcone, K. T. Gobeske, N. Petersen, J. Schindler, L. Sansing, E. J. Gilmore, D. Y. Hwang, J. A. Kim, A. Malhotra, G. Sze, M. S. Rosen, W. T.

Kimberly, K. N. Sheth, Portable, bedside, low-field magnetic resonance imaging for evaluation of intracerebral hemorrhage. *Nat. Commun.* **12**, 5119 (2021).

25. S. A. Parker, R. Bowry, T.-C. Wu, E. A. Noser, K. Jackson, L. Richardson, D. Persse, J. C. Grotta, Establishing the first mobile stroke unit in the United States. *Stroke* **46**, 1384–1391 (2015).

26. G. I. Ogbole, A. O. Adeyomoye, A. Badu-Peprah, Y. Mensah, D. A. Nzeh, Survey of magnetic resonance imaging availability in West Africa. *Pan Afr. Med. J.* **30**, 240 (2018).

27. R. Brouns, R. Sheorajpanday, J. Kunnen, D. De Surgeloose, P. P. De Deyn, Clinical, biochemical and neuroimaging parameters after thrombolytic therapy predict long-term stroke outcome. *Eur. Neurol.* **62**, 9–15 (2009).

28. A. Bruno, N. Shah, A. E. Akinwuntan, B. Close, J. A. Switzer, Stroke size correlates with functional outcome on the simplified modified rankin scale questionnaire. *J. Stroke Cerebrovasc. Disc.* **22**, 781–783 (2013).

29. A. Kruetzelmann, M. Köhrmann, J. Sobesky, B. Cheng, M. Rosenkranz, J. Röther, P. D. Schellinger, P. Ringleb, C. Gerloff, J. Fiehler, G. Thomalla, Pretreatment diffusion-weighted imaging lesion volume predicts favorable outcome after intravenous thrombolysis with tissue-type plasminogen activator in acute ischemic stroke. *Stroke* **42**, 1251–1254 (2011).

30. D. Z. Liu, F. Scalzo, S. Starkman, N. M. Rao, J. D. Hinman, D. Kim, L. K. Ali, J. L. Saver, A. R. Noorian, K. Ng, C. Liang, S. A. Sheth, B. Yoo, X. Liu, D. S. Liebeskind; UCLA-Jinling Neurovascular Imaging Research Collaborators, DWI lesion patterns predict outcome in stroke patients with thrombolysis. *Cerebrovasc. Dis.* **40**, 279–285 (2015).

31. H. Tei, S. Uchiyama, T. Usui, K. Ohara, Diffusion-weighted ASPECTS as an independent marker for predicting functional outcome. *J. Neurol.* **258**, 559–565 (2011).

32. A. J. Yoo, E. R. Barak, W. A. Copen, S. Kamalian, L. R. Gharai, M. A. Pervez, L. H. Schwamm, R. G. González, P. W. Schaefer, Combining acute diffusion-weighted imaging and mean transmit time lesion volumes with national institutes of health stroke scale score improves the prediction of acute stroke outcome. *Stroke* **41**, 1728–1735 (2010).

33. M. P. Lin, D. S. Liebeskind, Imaging of ischemic stroke. *Continuum (Minneap Minn)* **22**, 1399–1423 (2016).

34. B. C. V. Campbell, M. W. Parsons, Imaging selection for acute stroke intervention. *Int. J. Stroke* **13**, 554–567 (2018).

35. C. D. Morgan, M. Stephens, S. L. Zuckerman, M. S. Waitara, P. J. Morone, M. C. Dewan, J. Mocco, Physiologic imaging in acute stroke: Patient selection. *Interv. Neuroradiol.* **21**, 499–510 (2015).

36. J. B. Fiebach, P.D. Schellinger, A. Gass, T. Kucinski, M. Siebler, A. Villringer, P. Olkers, J.G. Hirsch, S. Heiland, P. Wilde, O. Jansen, J. Röther, W. Hacke, K. Sartor; Kompetenznetzwerk Schlaganfall B5, Stroke magnetic resonance imaging is accurate in hyperacute intracerebral hemorrhage - A multicenter study on the validity of stroke imaging. *Stroke* **35**, 502–506 (2004).

37. C. S. Kidwell, J. A. Chalela, J. L. Saver, S. Starkman, M. D. Hill, A. M. Demchuk, J. A. Butman, N. Patronas, J. R. Alger, L. L. Latour, M. L. Luby, A. E. Baird, M. C. Leary, M. Tremwel, B. Ovbiagele, A. Fredieu, S. Suzuki, J. P. Villablanca, S. Davis, B. Dunn, J. W. Todd, M. A. Ezzeddine, J. Haymore, J. K. Lynch, L. Davis, S. Warach, Comparison of MRI and CT for detection of acute intracerebral hemorrhage. *JAMA* **292**, 1823–1830 (2004).

38. H. M. Algethamy, M. Alzawahmah, G. B. Young, S. M. Mirsattari, Added value of MRI over CT of the brain in intensive care unit patients. *Can. J. Neurol. Sci.* **42**, 324–332 (2015).

39. P. D. Schellinger, O. Jansen, J. B. Fiebach, S. Heiland, T. Steiner, S. Schwab, O. Pohlers, H. Ryssel, K. Sartor, W. Hacke, Monitoring intravenous recombinant tissue plasminogen activator thrombolysis for acute ischemic stroke with diffusion and perfusion MRI. *Stroke* **31**, 1318–1328 (2000).

40. M. Sarracanie, C. D. LaPierre, N. Salameh, D. E. J. Waddington, T. Witzel, M. S. Rosen, Low-cost high-performance MRI. *Sci. Rep.* **5**, 15177 (2015).

41. Y. Liu, A. T. L. Leong, Y. Zhao, L. Xiao, H. K. F. Mak, A. C. O. Tsang, G. K. K. Lau, G. K. K. Leung, E. X. Wu, A low-cost and shielding-free ultra-low-field brain MRI scanner. *Nat. Commun.* **12**, 7238 (2021).

42. T. Rearick, G. L. Charvat, M. S. Rosen, J. M. Rothberg, U.S. Patent US9797971B2 (2017).

43. K. Chang, A. L. Beers, H. X. Bai, J. M. Brown, K. I. Ly, X. Li, J. T. Senders, V. K. Kavouridis, A. Boaro, C. Su, W. L. Bi, O. Rapalino, W. Liao, Q. Shen, H. Zhou, B. Xiao, Y. Wang, P. J. Zhang, M. C. Pinho, P. Y. Wen, T. T. Batchelor, J. L. Boxerman, O. Arnaout, B. R. Rosen, E. R. Gerstner, L. Yang, R. Y. Huang, J. Kalpathy-Cramer, Automatic assessment of glioma burden: A deep learning algorithm for fully automated volumetric and bidimensional measurement. *Neuro Oncol.* **21**, 1412–1422 (2019).

44. B. Zhu, J. Z. Liu, S. F. Cauley, B. R. Rosen, M. S. Rosen, Image reconstruction by domain-transform manifold learning. *Nature* **555**, 487–492 (2018).

45. M. Ebinger, I. Galinovic, M. Rozanski, P. Brunecker, M. Endres, J. B. Fiebach, Fluid-attenuated inversion recovery evolution within 12 hours from stroke onset a reliable tissue clock? *Stroke* **41**, 250–255 (2010).

46. M. G. Lansberg, V. N. Thijs, M. W. O'Brien, J. O. Ali, A. J. de Crespigny, D. C. Tong, M. E. Moseley, G. W. Albers, Evolution of apparent diffusion coefficient, diffusion-weighted, and T2-weighted signal intensity of acute stroke. *Am. J. Neuroradiol.* **22**, 637–644 (2001).

47. L. H. Schwamm, W. J. Koroshetz, A. G. Sorensen, B. Wang, W. A. Copen, R. Budzik, G. Rordorf, F. S. Buonanno, P. W. Schaefer, R. G. Gonzalez, Time course of lesion development in patients with acute stroke - Serial diffusion- and hemodynamic-weighted magnetic resonance imaging. *Stroke* **29**, 2268–2276 (1998).

48. G. Thomalla, P. Rossbach, M. Rosenkranz, S. Siemonsen, A. Krützelmann, J. Fiehler, C. Gerloff, Negative fluid-attenuated inversion recovery imaging identifies acute ischemic stroke at 3 hours or less. *Ann. Neurol.* **65**, 724–732 (2009).

49. L. M. Allen, A. N. Hasso, J. Handwerker, H. Farid, Sequence-specific MR imaging findings that are useful in dating ischemic stroke. *Radiographics* **32**, 1297–1299 (2012).

50. S. Dandamudi, J. D. Collins, J. C. Carr, P. Mongkolwat, A. A. Rahsepar, T. T. Tomson, N. Verma, R. Arora, A. B. Chicos, S. S. Kim, A. C. Lin, R. S. Passman, B. P. Knight, The safety of cardiac and

thoracic magnetic resonance imaging in patients with cardiac implantable electronic devices. *Acad. Radiol.* **23**, 1498–1505 (2016).

51. E. T. Martin, J. A. Coman, F. G. Shellock, C. C. Pulling, R. Fair, K. Jenkins, Magnetic resonance imaging and cardiac pacemaker safety at 1.5-Tesla. *J. Am. Coll. Cardiol.* **43**, 1315–1324 (2004).

52. S. Nazarian, R. Hansford, A. A. Rahsepar, V. Weltin, D. McVeigh, E. Gucuk Ipek, A. Kwan, R. D. Berger, H. Calkins, A. C. Lardo, M. A. Kraut, I. R. Kamel, S. L. Zimmerman, H. R. Halperin, Safety of magnetic resonance imaging in patients with cardiac devices. *N. Engl. J. Med.* **377**, 2555–2564 (2017).

53. T. Sommer, W. Bauer, K. Fischbach, C. Kolb, R. Luechinger, U. Wiegand, J. Lotz, I. Eitel, M. Gutberlet, H. Thiele, H. H. Schild, M. Kelm, H. H. Quick, J. Schulz-Menger, J. Barkhausen, D. Bänsch, MR imaging in patients with cardiac pacemakers and implantable cardioverter defibrillators. *Rofo* **189**, 204–217 (2017).

54. L. M. Broche, P. J. Ross, G. R. Davies, M. J. MacLeod, D. J. Lurie, A whole-body Fast Field-Cycling scanner for clinical molecular imaging studies. *Sci. Rep.* **9**, 10402 (2019).

55. J. C. Grotta, J.-M. Yamal, S. A. Parker, S. S. Rajan, N. R. Gonzales, W. J. Jones, A. W. Alexandrov, B. B. Navi, M. Nour, I. Spokoyny, J. Mackey, D. Persse, A. P. Jacob, M. Wang, N. Singh, A. V. Alexandrov, M. E. Fink, J. L. Saver, J. English, N. Barazangi, P. L. Bratina, M. Gonzalez, B. D. Schimpf, K. Ackerson, C. Sherman, M. Lerario, S. Mir, J. Im, J. Z. Willey, D. Chiu, M. Eisshofer, J. Miller, D. Ornelas, J. P. Rhudy, K. M. Brown, B. M. Villareal, M. Gausche-Hill, N. Bosson, G. Gilbert, S. Q. Collins, K. Silnes, J. Volpi, V. Misra, J. McCarthy, T. Flanagan, C. P. V. Rao, J. S. Kass, L. Griffin, N. Rangel-Gutierrez, E. Lechuga, J. Stephenson, K. Phan, Y. Sanders, E. A. Noser, R. Bowry, Prospective, multicenter, controlled trial of mobile stroke units. *N. Engl. J. Med.* **385**, 971–981 (2021).

56. U.S. Food and Drug Administration, Enforcement policy for imaging systems during the coronavirus disease 2019 (COVID-19) public health emergency: Guidance for industry and food and drug administration staff (U.S. Food and Drug Administration, 2020).

57. S. Rangaraju, D. Haussen, R. G. Nogueira, F. Nahab, M. Frankel, Comparison of 3-month stroke disability and quality of life across modified rankin scale categories. *Interv. Neurol.* **6**, 36–41 (2017).

58. E. K. Gibbons, P. Le Roux, S. S. Vasanawala, J. M. Pauly, A. B. Kerr, Body diffusion weighted imaging using non-CPMG fast spin echo. *IEEE Trans. Med. Imaging* **36**, 549–559 (2017).

59. P. Le Roux, G. McKinnon, Y. F. Yen, B. Fernandez, Realignment capability of the nCPMG sequence. *J. Magn. Reson.* **211**, 121–133 (2011).

60. D. O. Walsh, A. F. Gmitro, M. W. Marcellin, Adaptive reconstruction of phased array MR imagery. *Magn. Reson. Med.* **43**, 682–690 (2000).

61. R. B. Buxton, The diffusion sensitivity of fast steady-state free precession imaging. *Magn. Reson. Med.* **29**, 235–243 (1993).

62. E. X. Wu, R. B. Buxton, Effect of diffusion on the steady-state magnetization with pulsed field gradients. *J. Magn. Reson.* **90**, 243–253 (1990).

63. E. E. Sigmund, D. Gutman, Diffusion-weighted imaging of the brain at 7 T with echo-planar and turbo spin echo sequences: Preliminary results. *Magn. Reson. Imaging* **29**, 752–765 (2011).

64. T. G. Reese, O. Heid, R. M. Weisskoff, V. Wedeen, Reduction of eddy-current-induced distortion in diffusion MRI using a twice-refocused spin echo. *Magn. Reson. Med.* **49**, 177–182 (2003).
